# Quantification of Signaling Lipids by Nano-Electrospray Ionization Tandem Mass Spectrometry (Nano-ESI MS/MS)

**DOI:** 10.3390/metabo2010057

**Published:** 2012-01-16

**Authors:** Mathias Haag, Angelika Schmidt, Timo Sachsenheimer, Britta Brügger

**Affiliations:** 1 Heidelberg University Biochemistry Center, University of Heidelberg, Heidelberg, Germany; Email: mathias.haag@bzh.uni-heidelberg.de (M.H.); timo.sachsenheimer@bzh.uni-heidelberg.de (T.S.); 2 Tumorimmunology Program, Division of Immunogenetics (D030), German Cancer Research Center (DKFZ), Heidelberg, Germany; Email: a.schmidt@dkfz.de

**Keywords:** T cell signaling, signaling lipids, diacylglycerol, phosphoinositides, lipidomics, nano-electrospray ionization tandem mass spectrometry

## Abstract

Lipids, such as phosphoinositides (PIPs) and diacylglycerol (DAG), are important signaling intermediates involved in cellular processes such as T cell receptor (TCR)-mediated signal transduction. Here we report identification and quantification of PIP, PIP_2_ and DAG from crude lipid extracts. Capitalizing on the different extraction properties of PIPs and DAGs allowed us to efficiently recover both lipid classes from one sample. Rapid analysis of endogenous signaling molecules was performed by nano-electrospray ionization tandem mass spectrometry (nano-ESI MS/MS), employing lipid class-specific neutral loss and multiple precursor ion scanning for their identification and quantification. Profiling of DAG, PIP and PIP_2_ molecular species in primary human T cells before and after TCR stimulation resulted in a two-fold increase in DAG levels with a shift towards 1-stearoyl-2-arachidonoyl-DAG in stimulated cells. PIP_2_ levels were slightly reduced, while PIP levels remained unchanged.

## 1. Introduction

Diacylglycerols (DAGs) and phosphoinositides (PIPs) are low abundant lipid classes in cellular membranes whose abundance is temporally and spatially tightly regulated. For the bio-synthesis of PIPs, the precursor phosphatidylinositol (PI) needs to be transported from the ER to different organelles, where PIPs are metabolically generated and interconverted by the enzymatic action of several kinases and phosphatases [1,2,3]. Dependent on the position and extent of phosphorylation, seven distinct phosphoinositide classes are known: PI(3)P, PI(4)P, PI(5)P, PI(3,4)P_2_, PI(3,5)P_2_, PI(4,5)P_2_ and PI(3,4,5)P_3_, with PI(4)P and PI(4,5)P_2_ being the most abundant forms. Some phosphoinositide classes can be regarded as organelle markers since they are predominantly synthesized and located at specific membranes, such as PI(3)P at early endosomes, PI(4)P at the Golgi complex and PI(4,5)P_2_ and PI(3,4,5)P_3_ at the plasma membrane [4]. The phosphorylated headgroup of PIPs binds to effector proteins and thereby triggers signaling and activation processes. Several domains have been described to specifically interact with different types of PIPs. These domains include pleckstrin homology (PH), phagocyte oxidase (PX), epsin N-terminal homology (ENTH), C2 and FYVE zinc finger domains [4,5]. For example, at the trans-Golgi network PI(4)P binds to a variety of proteins, such as epsinR, AP-1, FAPP1, FAPP2, OSPB and to CERT, indicating important functional roles of PIPs in vesicular transport [6]. Plasma membrane-located PI(4,5)P_2_ is involved in a plethora of processes, such as endo- and exocytosis, plasma membrane cytoskeleton interactions, and signal transduction [4].

Adaptive immunity requires the activation of T cells in order to accomplish the response towards a specific antigen. Stimulation of T cells is achieved by antigenic ligation of the T cell receptor (TCR) in the presence of costimulation [7,8]. Activated T cells produce e.g. cytokines, which are required for the modulation of other immune cells. The expression of many cytokines is triggered through the activation of transcription factors *via* intracellular signaling pathways induced by TCR ligation. These intracellular signaling cascades involve, among other factors, the activation of phospholipase C γ1 (PLCγ1), which cleaves PI(4,5)P_2_ to generate the second messengers DAG and inositol-1,4,5-triphosphate (IP_3_) [9]. DAG activates protein kinase C θ (PKCθ), a critical player of the NF-κB pathway, and contributes to AP-1 activation *via* Ras/ERK [9]. IP_3_ on the other hand triggers Ca^2+^-release from the ER leading to store-operated calcium entry and NFAT activation [10]. Ca^2+^ signals further contribute to NF-κB activation. Activation of all three transcription factors (AP-1, NF-κB and NFAT) is needed for the expression of certain cytokines, such as the Th1-type-cytokines interleukin-2 (IL-2) and interferon -γ (IFN-γ) [8].

To study signaling processes in T cells, which are accompanied by subtle changes in DAG and PIP_2_, sensitive methods for the identification and quantification of these lipids are required. Conventional approaches for the analysis of PIPs are based on metabolic labeling with myo-[3H]-inositol followed by TLC- or HPLC-analysis [11,12,13]. DAG is traditionally analyzed by the DAG kinase assay [14] or by GC-MS after chemical derivatization [15,16]. Nevertheless, these methods are time- and sample-consuming and, furthermore, show limitations in the resolution of lipid classes and lipid species.

The combination of ESI [17] and (tandem) mass spectrometry was an important progress in the field of structural and quantitative lipid analysis [18,19,20,21,22,23,24,25]. ESI together with lipid class-specific (multiple)-precursor and neutral loss scanning on tandem mass spectrometers enabled the identification and quantification of lipid classes and lipid species directly from crude lipid mixtures. Consequently, the concept of shotgun-lipidomics arose [26,27,28,29,30]. However, in the past, ESI required relatively high amounts of starting material since lipid extracts were infused at flow rates in the µL/min-range. The replacement of the ESI source by a nano-ESI source was an imperative step forward in terms of sample consumption, thus allowing the sensitive analysis of lipid extracts at flow rates in the nL/min-range [27,29,31,32,33,34,35,36].

Within the last years a variety of methods have been reported allowing the analysis of PIPs by mass spectrometry [37]. ESI-MS/MS has been applied for the identification [38] and structural elucidation of PIPs [39]. Quantification of PIPs was demonstrated by ESI-MS/MS after LC separation [40,41,42] or by direct infusion of lipid extracts [43]. However, there is currently no method available that facilitates the quantification of PIPs by nano-ESI-MS/MS. Furthermore, ESI-MS/MS has been demonstrated for DAG quantification after chromatographic separation [44] or by direct infusion after derivatization [45]. Quantification of positively charged DAG ammonium adducts by nano-ESI-MS/MS was recently demonstrated by neutral loss scanning [34] and, additionally, multiple precursor ion scanning (MPIS) was reported to be applicable for DAG quantification in positive ion mode [29].

Although a variety of methods for the mass spectrometric analysis of PIPs and DAG is currently available, all approaches consider both lipid classes separately with respect to their analysis. In this work, a method for the simultaneous identification and quantification of the signaling intermediates DAG, PIP and PIP_2_ is presented. The approach takes advantage of the different extraction properties of these structurally diverse lipid classes. By performing a two-step extraction, both lipid classes can be isolated from one sample at the same time. Nano-ESI MS/MS in combination with internal standards and lipid class-specific scanning was used for the identification and quantification of endogenous signaling lipids. As a proof of principle the method was applied to the profiling of DAG, PIP and PIP_2_ molecular species in primary human T cells before and after TCR stimulation.

## 2. Results

### 2.1. Extraction of PIPs

Due to their polar headgroups, PIPs are not sufficiently recovered from biological membranes by conventional extraction procedures such as Folch [46] or Bligh and Dyer [47]. Therefore, modified extraction protocols were introduced decades ago [48] and continuously optimized and applied for the analysis of PIPs [49,50,51,52]. A variety of these methods employ acidification of extraction solvents to protonate phosphate groups and thereby facilitate the disruption of ionic interactions with proteins. Furthermore, protonation supports partitioning into the organic phase, which increases the recovery of low abundant PIPs. In contrast to PIPs, DAGs, as neutral lipids, are efficiently extracted by conventional methods.

**Figure 1:**
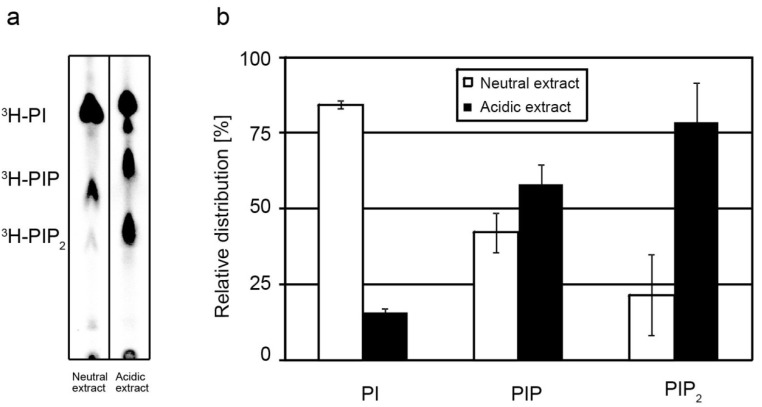
Recovery of endogenous PIPs. HeLa cells were metabolically labeled with myo-[3H]-inositol. Following a two-step lipid extraction, ^3^H-labeled inositol lipids were separated by TLC and visualized by autoradiography. Autoradiogram (**a**) and quantification (**b**) showing the distribution of ^3^H-PI and ^3^H-PIPs between neutral and acidic extracts. To avoid saturation of the detector, maximal 200 nCi were used for TLC analysis. For quantification, the spot intensities were extrapolated to determine the absolute amounts of PI and PIPs in lipid extracts. Data represent mean values ± SD of four independent experiments.

To make the chemically diverse lipids DAGs and PIPs accessible for simultaneous mass spectrometric analysis, a previously described two-step extraction procedure for the selective enrichment of PIPs was performed [52]. With the first extraction apolar lipids such as DAGs are extracted with a mixture of chloroform/methanol (neutral extraction). Subsequently, negatively charged PIPs, which to a substantial part reside in the membrane, are recovered by an acidic extraction using a mixture of chloroform/methanol/HCl. In order to determine the extraction behavior of endogenous PIPs, HeLa cells were labeled with myo-[3H]-inositol and subjected to a two-step lipid extraction. ^3^H-inositol-labeled lipids in neutral and acidic extracts were separated by TLC and visualized by autoradiography (Figure 1a). While most of ^3^H-PI was recovered with the neutral extraction, the phosphoinositides ^3^H-PIP and ^3^H-PIP_2_ were predominantly present in the acidic extract. Quantification of the spot intensities revealed that PI was recovered to ~84% in the neutral extract, and to ~16% in the acidic extract (Figure 1b). In contrast, PIP and PIP_2_ were enriched with ~58% and ~79% in the acidic extract. It is of note that we observed a different extraction behavior of endogenous PIPs compared to exogenously added PIP and PIP_2_ standards. Whereas endogenous PIP and PIP_2_ were protected to more than 50% from the neutral extraction, exogenously added PIP and PIP_2_ standards were recovered already to ~50% in the neutral extraction (data not shown). The different extraction behavior of endogenous and exogenous PIPs is an important issue when considering at what step during the extraction process PIP and PIP_2_ standards should be spiked to the sample to allow for correct mass spectrometric quantification.

Taken together, the labeling experiments demonstrated that the two-step extraction provides an easy and fast approach to enrich PIPs from cellular membranes. Importantly, the initial neutral extraction recovers DAG and depletes the membrane pellet from abundant lipids such as phosphatidylcholine (PC), phosphatidylethanolamine and PI, which otherwise could cause ion suppression and thus reduced ionization efficiency of PIPs during mass spectrometric analysis.

### 2.2. Mass Spectrometric Characterization of DAGs and PIPs

**Figure 2:**
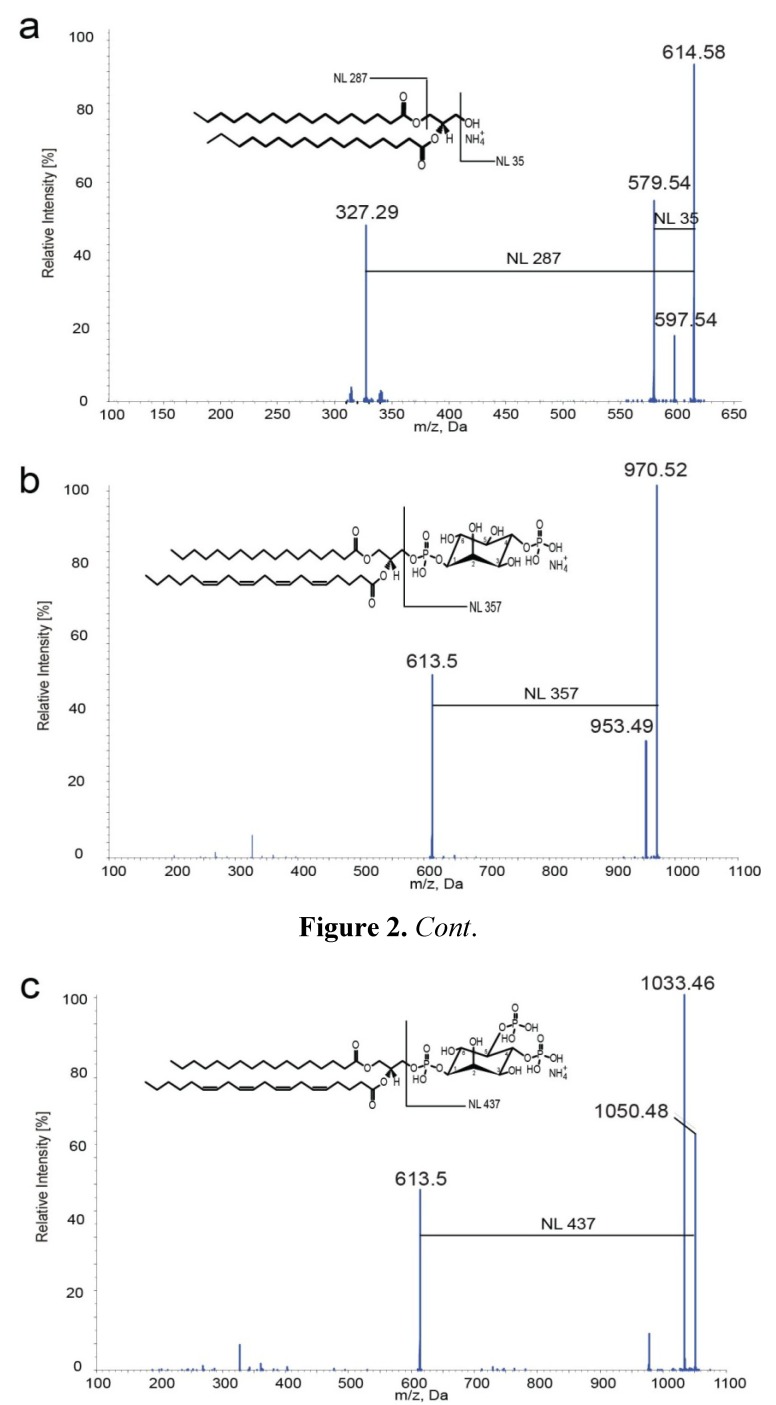
Fragmentation of DAG, PIP and PIP_2_ ammonium adducts. Product ion analyses of DAG 17:0/17:0 (a), PI(4)P 17:0/20:4 (b) and PI(4,5)P_2_ 17:0/20:4 (c). Characteristic fragment ions and corresponding neutral losses are indicated.

It was previously shown that DAGs form positively charged ammonium adducts that display characteristic fragment ions upon dissociation when analyzed by nano-ESI MS/MS [34]. To analyze the fragmentation behavior of DAG with the instrumentation used in this study, a commercially available DAG species with fatty acid composition 17:0/17:0 (DAG 34:0) was analyzed in the presence of ammonium acetate. Collision-induced dissociation (product ion analysis) of the ammoniated precursor (*m/z* 614.6) resulted in a fragment pattern typically observed for DAGs [34] (Figure 2a). Three characteristic product ions were detected at *m/z* 597.54, *m/z* 579.54 and *m/z* 327.29. The fragment at *m/z* 579.54 results from the dissociation of H_2_O + NH_3_, leading to a characteristic neutral loss of 35 Da (NL 35) [34]. The fragment at *m/z* 327.29 reflects the neutral loss of the fatty acid 17:0 together with ammonia (NL 287 = RCOOH + NH_3_) [34], leading to the positively charged monoacylglycerol-H_2_O fragment MAG-H_2_O 17:0. In contrast to previous observations [34], a weak loss of ammonia (NH_3_, neutral loss of 17 Da) was detected, leading to a protonated DAG molecule at *m/z* 597.54. Nevertheless, the presence of this ion depended on the collision energy (CE) as it disappeared at higher CE offsets (data not shown).

To characterize PIPs, the ionization and fragmentation behavior of two commercially available phosphoinositides, PI(4)P and PI(4,5)P_2_, both with fatty acid composition 17:0/20:4 (37:4) was investigated. When analyzing in positive ion mode in the presence of 25 mM ammonium acetate in chloroform/methanol/H_2_O (1:1:0.05), PI(4)P 37:4 and PI(4,5)P_2_ 37:4 showed distinct MS signals at *m/z* 970.5 and at *m/z* 1050.5, respectively (data not shown). The mass differences of 17 Da compared to the calculated, nominal masses of *m/z* 953.51 for PI(4)P 37:4 and *m/z* 1033.48 for PI(4,5)P_2_ 37:4 indicated that both molecules appeared as ammonium adducts (+NH_4_^+^). Collision-induced dissociation (product ion mode) of the ammoniated precursor molecules resulted in the formation of two prominent fragments for PIP and PIP_2_, respectively (Figure 2b,c). The fragment ions at *m/z* 953.5 and *m/z* 613.5 in the product ion spectra of PI(4)P 37:4 (Figure 2b) correspond to neutral losses of ammonia (17 Da) and inositoldiphosphate + NH_3_ (357 Da). PI(4,5)P_2_ 37:4 showed a similar fragmentation pattern, resulting in ions at *m/z* 1033.46 (neutral loss of ammonia) and *m/z* 613.5 (neutral loss of inositoltriphosphate + NH_3_ (437 Da)) (Figure 2c).

**Figure 3:**
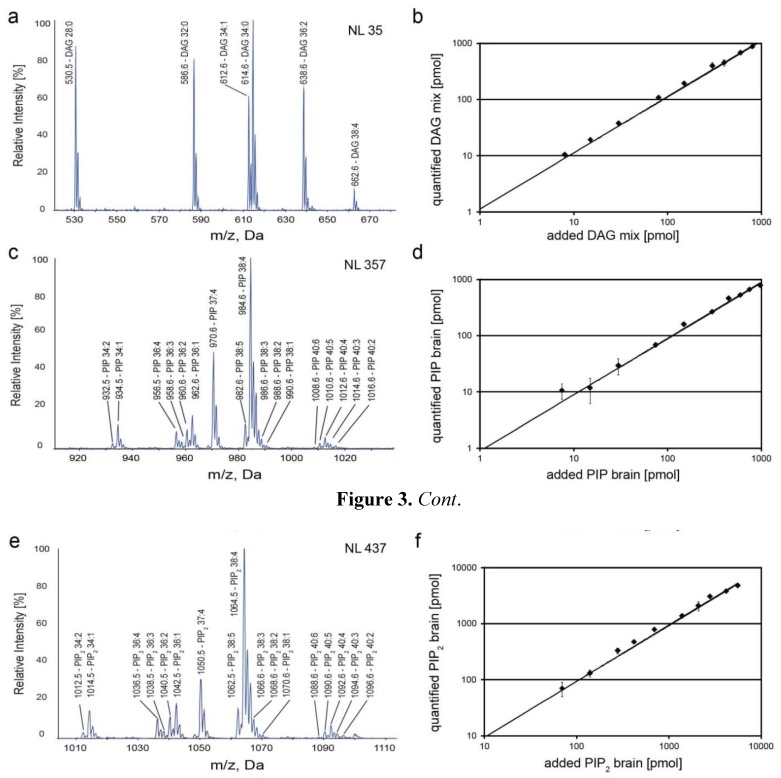
Identification of DAG, PIP and PIP_2_ by scanning for headgroup-specific neutral losses (a,c,e). (a) Scanning for neutral losses of 35 Da enables identification of DAG species. Scanning for neutral losses of 357 Da and 437 Da allows identification of PIP brain (c) and PIP_2_ brain (e) species, respectively. Linearity of DAG, PIP and PIP_2 _ quantification (b,d,f); (b) The standard DAG 17:0/17:0 was spiked into an equimolar mixture of DAG 14:0/14:0, DAG 16:0/16:0, DAG 16:0/18:1, DAG 18:1/18:1 and DAG 18:0/20:4. Quantification was performed by MPIS; (d) The standard PI(4)P 17:0/20:4 was spiked into extracts of PIP brain. Quantification was performed by scanning for neutral loss of 357 Da; (f) PIP_2_ brain species were quantified by scanning for neutral loss of 437 Da using PI(4,5)P_2_ 17:0/20:4 as standard. Data represent mean ± SD of three independent experiments.

We next tested whether using DAG 17:0/17:0 and PIP/PIP_2_ 17:0/20:4 as internal standards and scanning for lipid class specific fragment ions enables identification of DAG, PIP and PIP_2_ molecular species. To this end, dilution series of equimolar mixtures of “endogenous-like” DAG species (DAG 14:0/14:0, DAG 16:0/16:0, DAG 16:0/18:1, DAG 18:1/18:1 and DAG 18:0/20:4), PIP and PIP_2_ brain extracts were analyzed together with constant spike amounts of DAG 17:0/17:0, PI(4)P 17:0/20:4 and PI(4,5)P_2_ 17:0/20:4. The mixtures were analyzed by headgroup-specific scanning for neutral losses of 35 Da (DAG), 357 Da (PIP) and 437 Da (PIP_2_), respectively. As a result, neutral loss scanning allowed rapid identification of endogenous signaling lipids together with the internal standards with response factors of 1 (Figure 3a,c,e). However, we observed that the detection sensitivity of polyunsaturated DAG 38:4 was drastically reduced when compared to DAG species with a higher degree of saturation (Figure 3a). Thus, although NL 35 scanning allows rapid identification of DAG species, accurate quantification of endogenous DAG requires an alternative approach. DAG mixtures were therefore analyzed by multiple precursor ion scanning (MPIS) [33] for DAG-specific MAG-H_2_O ions. As displayed in Figure 3b MPIS allowed the correct quantification of all endogenous DAG species, which can be quantified over a dynamic range of at least two orders of magnitude. Importantly, in contrast to quantification of DAG species by NL 35 scanning, MPIS was not significantly affected by fatty acid chain length and degree of unsaturation, *i.e.*, number of double bonds (Supplementary Figure 1), allowing to use only one standard for quantitation, as shown before by [33,53]. Likewise, neutral loss scanning allowed accurate quantification of PIP and PIP_2_ over a broad dynamic range (Figure 3d,f). The limit of quantification (LOQ) was determined to 10–15 pmol, both for DAG and PIP, and to 70 pmol for PIP_2_. This result is in line with previous observations [43] and is likely due to a lower ionization efficiency of PIP_2_.

Taken together, the mass spectrometric investigation of DAG, PIP and PIP_2_ species showed that both lipid classes can be detected as ammonium adducts, facilitating their mass spectrometric analysis in positive ion mode. Collision-induced dissociation confirmed the formation of DAG-specific product ions as described [34] and revealed lipid class-specific fragments of PIP and PIP_2_ that allow their rapid identification.

### 2.3. Identification and Quantification of DAG, PIP and PIP_2_ in Human T Cells

**Figure 4:**
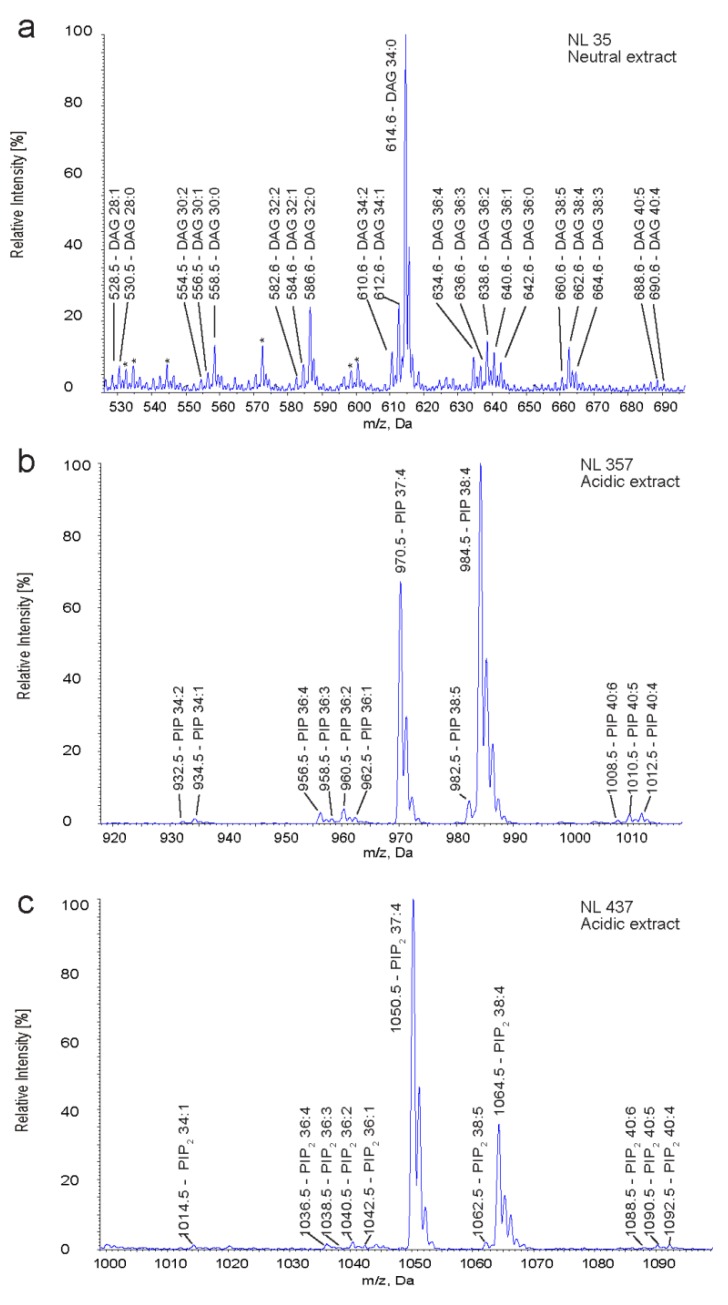
Identification of DAG, PIP and PIP_2_ molecular species in human T cells by neutral loss scanning for lipid class-specific fragments. (**a**) DAG species identified by scanning for neutral losses of 35 Da in neutral extracts. Asterisks indicate unidentified peaks. (**b**) PIP and (**c**) PIP_2_ species identified by scanning for neutral losses of 357 Da (PIP) and 437 Da (PIP_2_) in acidic extracts.

The above described two-step extraction procedure together with lipid class-specific multiple precursor ion and neutral loss scanning was performed for the simultaneous identification and quantification of molecular species of DAG, PIP and PIP_2_ in primary human CD4^+^CD25^−^ T cells. DAG, PIP and PIP_2_ with non-endogenous fatty acid compositions (Figure 2) were used as internal standards to enable quantification of endogenous lipids. DAG 17:0/17:0 was spiked prior neutral extraction, whereas PI(4)P 17:0/20:4 and PI(4,5)P_2_ 17:0/20:4 were spiked prior to the acidic extraction. Scanning for DAG-specific neutral losses of 35 Da was performed to reveal a DAG species profile in T cells (Figure 4a). 20 DAG species were identified. The internal standard DAG 17:0/17:0 was detected at *m/z* 614.6, which is isobaric to endogenous DAG 16:0/18:0 and thus leads to overlapping of both species, when applying headgroup-specific scanning for neutral losses of 35 Da. In addition, NL 35 scanning underestimates polyunsaturated DAG species such as DAG 38:4 as compared to the internal standard DAG 17:0/17:0 (Figure 3a and Supplementary Figure 1). We therefore performed MPIS of DAG-specific MAG-H_2_O fragments, which allowed unperturbed quantification of all molecular DAG species irrespective of their saturation state (Supplementary Figure 1). The most abundant species detected were DAG 34:1, DAG 34:2, DAG 36:1 and DAG 38:4 comprising ~13, ~12, ~11 and ~11 mol% of all identified DAG species, respectively (Table 1). The amount of total DAG (sum of all species) was quantified to 8 ± 2.5 fmol/pmol PC (n = 3). Assuming that PC accounts for ~45% of phospholipids in lymphocytes [54], DAG comprises ~0.36 mol% of phospholipids in T cells.

For the identification of PIP and PIP_2_ species, neutral loss scanning was performed in acidic extracts of T cells. The neutral loss spectra NL 357 Da and NL 437 Da display endogenous PIP and PIP_2_ species and the internal standards used (Figure 4b,c). Scanning for neutral losses of 357 Da enabled the identification of 11 PIP species, with PIP 38:4 being the most abundant ion (accounting for ~82 mol% of total PIP) (Table 1). Scanning for neutral losses of 437 revealed 10 PIP_2_ species, with PIP_2_ 38:4 as most abundant ion accounting for ~70 mol% of total PIP_2_. For absolute quantification of PIP and PIP_2_, the raw values were corrected for losses during the neutral extraction (factor of 1.72 for PIP and 1.27 for PIP_2_). The absolute amount of PIP and PIP_2_ in T cells was quantified to 4.8 ± 1.7 and 5 ± 1.8 fmol/pmol PC (n = 3), respectively. With PC accounting for ~45% of phospholipids [54], the amount of PIP and PIP_2_ comprises each around 0.22 mol% of total phospholipid.

**Table 1:** DAG, PIP and PIP_2_ molecular species identified in lipid extracts of primary human T cells. Identification of DAG species in neutral extracts was done by NL 35 scanning, quantification was done by MPIS. Identification and quantification of PIP and PIP_2_ species in acidic extracts was achieved by NL 357 and NL 437 scanning, respectively. *m/z* values correspond to ammonium adducts (NH_4_^+^). Mol% indicates the distribution of lipid species within a given lipid class. Data represent mean values ± SD of three independent analyses.

Species	DAG		PIP		PIP_2_	
	*m/z*	mol%	*m/z*	mol%	*m/z*	mol%
28:1	528.5	2.1 ± 0.6	-	-	-	-
28:0	530.5	0.5 ± 0.2	-	-	-	-
30:2	554.5	1.0 ± 0.1	-	-	-	-
30:1	556.5	3.1 ± 0.8	-	-	-	-
30:0	558.5	1.6 ± 0.6	-	-	-	-
32:2	582.6	1.9 ± 0.5	-	-	-	-
32:1	584.6	5.4 ± 0.9	-	-	-	-
32:0	586.6	3.9 ± 0.6	-	-	-	-
34:2	610.6	11.8 ± 2.5	932.5	0.5 ± 0.2	-	-
34:1	612.6	12.6 ± 1.8	934.5	1.1 ± 0.4	1014.5	3.6 ± 2.8
34:0	614.6	3.2 ± 0.8	-	-	-	-
36:4	634.6	6.4 ± 1.9	956.5	3.6 ± 1.5	1036.5	5.5 ± 3.1
36:3	636.6	6.0 ± 4.1	958.5	0.8 ± 0.1	1038.5	2.2 ± 2.2
36:2	638.6	5.5 ± 0.8	960.5	2.4 ± 0.5	1040.5	4.2 ± 0.3
36:1	640.6	11.2 ± 1.1	962.5	0.9 ± 0.2	1042.5	3.3 ± 2.3
36:0	642.6	2.4 ± 0.4	-	-	-	-
38:5	660.6	3.3 ± 0.6	982.5	5.0 ± 0.6	1062.5	5.5 ± 0.9
38:4	662.6	10.8 ± 2.2	984.5	82.2 ± 1.5	1064.5	70.0 ± 11.1
38:3	664.6	2.1 ± 0.9	-	-	-	-
40:6	-	-	1008.5	0.6 ± 0.3	1088.5	1.4 ± 0.8
40:5	688.6	2.7 ± 0.4	1010.5	1.6 ± 0.3	1090.5	2.1 ± 1.8
40:4	690.6	2.3 ± 0.3	1012.5	1.5 ± 0.2	1092.5	2.2 ± 1.5

### 2.4. Effect of TCR Stimulation on DAG, PIP and PIP_2_ in Human T Cells

**Figure 5:**
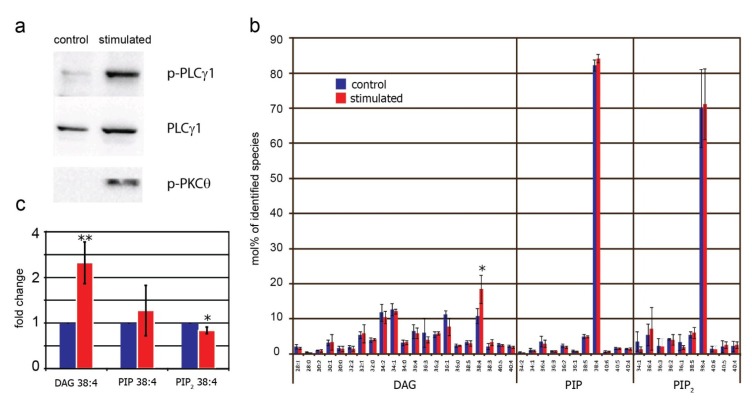
Effect of TCR stimulation on DAG, PIP and PIP_2_ levels in T cells. Primary human T cells were stimulated for 5 min followed by a two-step lipid extraction. DAG was analyzed by MPIS in neutral extracts, PIP and PIP_2_ were analyzed by scanning for neutral losses of 357 Da and 437 Da in acidic extracts. (**a**) Phosphorylation of PLCγ1 (p-PLCγ1) and PKCθ (p-PKCθ) confirmed the activation of intracellular signaling pathways; (**b**) Effect of TCR stimulation on the relative distribution of DAG, PIP and PIP_2_ molecular species identified in T cells. Mol% indicates the distribution of lipid species within a given lipid class; (**c**) Effect of TCR stimulation on the major species DAG 38:4, PIP 38:4 and PIP_2_ 38:4. Unstimulated T cells of the same donor were set at 1 and fold-change after stimulation was calculated. Data represent mean values ± SD of three independent analyses with different donors. * *p* < 0.05, ** *p* < 0.01.

To test whether the described approach is suitable to monitor transient changes in the overall levels of PIP, PIP_2_ and DAG in cells, lipid extracts from T cells were analyzed before and after TCR stimulation. To this end, primary human T cells were stimulated for 5 min using agonistic anti-CD3 antibodies. Co-stimulation was achieved with anti-CD28 antibodies, and cross-linking antibodies were added to enhance stimulation. Phosphorylation of PLCγ1 and PKCθ confirmed the activation of intracellular signaling pathways upon TCR ligation (Figure 5a). Unstimulated and stimulated T cells were subjected to a two-step lipid extraction. DAG quantification was performed in neutral extracts, PIP and PIP_2_ levels were quantified in acidic extracts. As depicted in Figure 5b, TCR stimulation significantly increased the level of DAG 38:4. Detection of an elevated level of DAG 38:4 is in accordance with the fact that PIP_2_ 38:4 is the main source for PLCγ1-generated DAG in activated T cells. In contrast to DAG, TCR stimulation did not affect the species profiles of PIP and PIP_2_ (Figure 5b). Quantification of the major species PIP_2_ 38:4, PIP 38:4, and DAG 38:4 confirmed elevated levels of DAG 38:4, which was accompanied by a slight but significant reduction of PIP_2_ 38:4. The amount of PIP 38:4 did not significantly change upon T cell activation (Figure 5c). Interestingly, the increase in DAG 38:4 was not accompanied by an equivalent decrease of PIP_2_ 38:4, which one might expect due to PLCγ1-induced conversion of PIP_2_ to DAG. This effect may indicate that upon TCR stimulation PIP_2_ 38:4 is rapidly re-synthesized by phosphorylation of its precursor PIP 38:4 and/or by dephosporylation of PIP_3_ 38:4.

## 3. Discussion

The purpose of this study was to establish a workflow for the simultaneous and rapid identification and quantification of the signaling lipids DAG, PIP and PIP_2_ by nano-ESI MS/MS. These lipid classes are intimately involved in early signaling processes, as PIP_2_ is rapidly converted to DAG by action of PLCγ during T cell activation [9], and PIP_2_ is re-synthesized by phosphorylation of PIP [55]. Therefore, monitoring subtle changes in the abundance of these lipids is of great importance in order to understand the mechanisms of lipid-mediated signaling and potential alterations in diseases. A variety of methods for the mass spectrometric analysis of PIPs [37] and DAG [34,44,45] are currently available. All methods, however, consider both lipid classes separately with respect to their analysis. Capitalizing on different extraction properties of structurally diverse lipids [27], we were able to selectively enrich DAG and PIPs by a two-step extraction [52] allowing their simultaneous mass spectrometric profiling from one sample.

DAG can be quantitatively recovered by neutral extraction procedures, whereas PIPs are protected substantially from the neutral extraction. Different observations were made regarding the extent of PIP and PIP_2_ recovery during neutral extraction. Whereas Milne *et al* did not detect any PIP_2_ in neutral extracts of RAW 264.7 cells [38], Gray *et al* observed a partial extraction of PIP (26%) and PIP_2_ (6%) in astrocytoma cells [52]. We tested the extraction efficiency of PIPs in HeLa cells and found even higher losses of PIP (42%) and PIP_2_ (21%) during the neutral extraction. These discrepancies in the extraction behavior are most likely due to the different cell types used.

The mass spectrometric analysis of commercially available DAG, PIP and PIP_2_ species showed that these lipid classes can be detected as ammonium adducts, facilitating their mass spectrometric analysis in positive ion mode. Most of the available methods for the measurement of PIPs rely on the analysis in negative ion mode [38,39,40,41,43], which should be beneficial because of the negatively charged phosphate groups. However, we found that negatively charged PIPs showed a variable ionization behavior on ESI-MS/MS systems equipped with a nanospray source (data not shown). Consequently, it was not possible to create conditions that resulted in a constant stream of either singly or doubly charged PIPs. Negatively charged PIPs were rather fluctuating between singly and doubly charged states and thus did not allow for robust analysis. In contrast, positively charged ammonium adducts resulted in a stable ionization behavior of PIP and PIP_2_. PIP_3_ is an extremely low abundant lipid accounting for <0.002 mol% of phospholipids in mammalian cells [56], and we could not detect this lipid with the described approach. For the detection of PIP_3_, LC-MS based analyses using higher flow rates than nano-flow with [42] or without [40,41] headgroup modification were reported as alternative approaches with sufficient sensitivity.

The mass spectrometric analysis of DAG confirmed the formation of positively charged ammonium adducts that, upon collision-induced dissociation, display lipid class-specific fragments [34]. The neutral loss of fatty acid fragments of DAG species results in characteristic MAG-H_2_O ions that were chosen to perform DAG-specific MPIS as reported [29]. Although DAG species can be detected by scanning for neutral losses of 35 Da, quantification should be done by MPIS or neutral loss scanning of fatty acid-derived ions [34], as only these scanning procedures allow accurate quantification of DAG independent of the saturation state. In particular DAG 38:4 showed a drastically reduced NL 35 detection sensitivity. Most likely, this effect can be attributed to the polyunsaturated fatty acid 20:4, which might influence the fragmentation behavior of polyunsaturated DAG species. Further experiments are required to reveal additional information about the mass spectrometric dissociation behavior of DAG 38:4. A combination of MPIS and neutral loss scanning of fatty acid-derived ions also allows to analyze ether- and odd-numbered fatty acyl-DAGs. However, additional internal standards may be required for the absolute quantification of ether-DAGs since the mass spectrometric fragmentation behavior of ether-DAGs might differ from acyl-DAGs, such as previously observed for acyl-PE and alkenyl-PE [57].

Profiling of DAG and PIPs in T cells before and after TCR stimulation demonstrated that subtle changes in the abundances of signaling lipids can be monitored with the method described here. A 5 min stimulation of the TCR resulted in significantly increased levels of DAG 38:4, consistent with the activation of PLCγ1 and PKCθ. Furthermore, the level of PIP_2_ 38:4 was slightly but significantly reduced upon TCR stimulation. The increase in DAG 38:4 did not go along with a corresponding reduction of PIP_2_ 38:4 to the same extent. Two possibilities can be envisaged to explain this effect. First, re-synthesis of PIP_2_: This scenario is supported by slightly elevated levels of PIP 38:4, the precursor of PIP_2_ biosynthesis. Indeed, the synthesis rate of PIPs is known to be increased upon TCR stimulation [55]. Second, DAG might be generated from other lipid precursors than PI(4,5)P_2_. Although it is widely accepted that TCR stimulation triggers PLCγ1 that cleaves plasma membrane-located PI(4,5)P_2_, there is evidence that activation of Ras/MAPK pathways by DAG can also occur at the Golgi complex [58]. Thus, other lipid precursors might contribute to the production of DAG, such as PC that can be converted to DAG by the action of phospholipase D and PA phosphatase, enzymes that have previously been linked to TCR signaling [59]. To identify the source of DAG in activated T cells, the analysis of PIP_2_-derived inositol-phosphate intermediates is required in future studies. In addition, it will be important to monitor other metabolites that are affected during T cell activation, such as PA and PIP_3_ [9,60].

## 4. Experimental Section

*Lipids:* PI(4)P 17:0/20:4 (# LM-1901), PI(4,5)P_2_ 17:0/20:4 (# LM-1904), Brain PI(4)P (# 840045P), Brain PI(4,5)P_2_ (# 840046P), DAG 14:0/14:0 (# 800814C), DAG 16:0/16:0 (# 800816C), DAG 16:0/18:1 (# 800815C), DAG 18:1/18:1 (# 800811C), DAG 18:0/20:4 (# 800818C), PC 13:0/13:0 (# 850340C), PC 14:0/14:0 (# 850345C), PC 20:0/20:0 (# 850368C) and PC 21:0/21:0 (# 850370C) were purchased from Avanti Polar Lipids (Alabaster, USA). DAG 17:0/17:0 (# 32-1702) was obtained from Larodan (Malmö, Sweden). DAG and PC were dissolved in chloroform, PIP and PIP_2_ were dissolved in chloroform/methanol/H_2_O (1:1:0.2, v/v/v). The concentration of phospholipid solutions was determined by the phosphorous assay [61]. Lipid stocks were diluted to final concentrations of 1-10 µM. PIP_2_ mixtures were kept at concentrations >30 µM.

*Cell culture and myo*-[3H]-inositol labe*ling*: Labeling of HeLa cells was performed as described [11]. Briefly, HeLa cells (# ATCC CCL-2.1) were maintained in 10-cm dishes in DMEM (including 10% fetal calf serum, 100 µg/mL penicillin, 100 µg/mL streptavidin and 2 mM glutamine) in 5% CO_2_ at 37 °C in a humidified incubator. Cells were grown to ~50–60% confluency, the medium was replaced by inositol-free DMEM (including 10% fetal calf serum, 100 µg/mL penicillin, 100 µg/mL streptavidin and 2 mM glutamine) and myo-[3H]-inositol was added to a final concentration of 15 µCi/mL. The labeling time was typically 24–48 h until the cells reached levels of ~80–90% confluency.

*Phosphoinositide extraction from my*o-[3H]-inositol lab*eled HeLa cells:* Phosphoinositide extraction was performed as described [52]. Briefly, after transferring the cells (~ 5 × 10^6^) to ice and removal of the medium, cells were washed two times with 10 mL cold PBS. After adding 1.2 ml cold 0.5 M TCA, the cells were immediately scraped off the dish, transferred to an Eppendorf tube and vortexed for 10 sec followed by 5 min incubation on ice. After centrifugation (13,800 × g, 2 min at 4 °C), the supernatant was discarded and the pellet washed two times with 1 mL cold 5% TCA/1 mM EDTA. For neutral extraction, the pellet was resuspended in 1 mL chloroform/methanol (1:2, v/v) and incubated for 10 min at RT. The sample was vortexed for 30 s every 3 min. After precipitation (13,800 × g, 2 min at 4 °C), the supernatant was transferred to a new Eppendorf tube. For acidic extraction, the remaining pellet was resuspended in 750 µL chloroform/methanol/37% HCl (40:80:1, v/v/v) and incubated for 15 min at RT while vortexing the sample for 30 s every 5 min. After transferring the tube to ice, 250 µL cold chloroform and 450 µL cold 0.1 M HCl was added followed by 1 min vortexing and centrifugation (6,500 × g, 2 min at 4 °C). The bottom organic phase was transferred to a new tube. Before drying, ~1 vol% of the neutral and the acidic extract was subjected to scintillation counting to determine the radioactivity. Based on the sum of radioactivities in the neutral extract (100 vol%) and the acidic extract (100 vol%), a typical myo-[3H]-inositol incorporation of 0.5–1.5% was calculated compared to the starting labeling amount (150 µCi). The organic phases were dried and stored at −20 °C until further processing.

*Thin-layer chromatography (TLC) and autoradiography*: For TLC analysis, Silica 60 plates (Merck, Darmstadt) were covered with 1% (w/v) potassium oxalate and 2 mM EDTA in 50% methanol followed by drying for 15 min with a blow-dryer. Dried lipid extracts were dissolved in appropriate volumes (20–300 µL) of chloroform/methanol/H_2_O (1:1:0.2, v/v/v) to a final concentration of ~5–15 nCi/µL. 50–200 nCi were spotted on TLC-plates and dried (Note: to avoid saturation of the ^3^H detector, only a proportion of each extract was spotted on the plates). The plates were developed with chloroform/acetone/methanol/acetic acid/H_2_O (64:30:24:30:13, v/v/v/v/v). After drying, ^3^H-PI and ^3^H-PIPs were visualized for 24 h by the beta imager (Biospace, France). Data evaluation was performed with the β-vision+ software (Biospace, France). The spot activities (cpm) of ^3^H-PI and ^3^H-PIPs were used to determine the distribution of ^3^H-PI, ^3^H-PIP and ^3^H-PIP_2_ in neutral and acidic extracts.

*Preparation of primary human T cells and TCR stimulation*: Human peripheral blood leucocytes (PBLs) were purified from Buffy coats by Biocoll™ (Biochrom, Berlin, Germany) gradient centrifugation followed by plastic adherence to deplete monocytes. CD4^+^CD25^−^ conventional T cells (Tcons) were isolated by magnetic cell sorting (MACS) using the CD4-Isolation kit II and additionally depleted from CD25^+^-cells with 8 µL of anti-CD25 MACS beads per 10^7^ cells (Miltenyi Biotech). Tcons were rested overnight in X-Vivo 15 medium (Lonza) supplemented with 1% Glutamax. Tcons were taken up in pre-warmed X-Vivo 15 medium (37 °C) to ~10^7^ cells/mL and stimulated by addition of soluble anti-CD3 (0.2 µg/mL), anti-CD28 (2 µg/mL) and goat anti-mouse crosslinker (2 µg/mL) antibodies for indicated time periods at 37 °C/ 5% CO_2_. 5 × 10^6^ cells were used per sample. Stimulation was stopped by the addition of ice-cold MACS-buffer. After centrifugation (1,000 × g, 5 min at 4 °C), Tcons were washed with PBS and lysed in 0.6 mL 0.5 M TCA.

*Lipid extraction from primary human T cells*: Two-step lipid extraction was performed similar as described [52]. Briefly, T cell lysates were either immediately processed or snap frozen and stored at −80 °C until further processing. The storage time was restricted to a maximum of 2–3 weeks to minimize sample degradation. Prior to lipid extraction, TCA lysates were precipitated (13,800 × g, 2 min at 4 °C) and pellets washed two times with 1 mL cold 5% TCA/ 1 mM EDTA. After centrifugation (13,800 × g, 2 min at 4 °C), the supernatant was discarded and lipids were extracted in Eppendorf tubes. Prior to neutral extraction, 15–50 pmol DAG 17:0/17:0 and 250 pmol of an equimolar mixture of PC standards was spiked to the pellet and lipids were extracted with 750 µL chloroform/methanol (1:2, v/v) by incubating 10 min at RT and vortexing every 3 min for 30 s. After centrifugation (13,800 × g, 2 min at 4 °C), the supernatant was carefully removed from the pellet and transferred to a new tube. The supernatant was subjected to a second extraction after the addition of 250 µL chloroform and 450 µL water. Phase separation was induced by centrifugation (6,500 × g, 2 min at 4 °C) and the lower phase was transferred to a new tube. Dried lipids were dissolved in 10 mM ammonium acetate in methanol and subjected to mass spectrometry.

Prior to acidic extraction of PIPs, 15–25 pmol PI(4)P 37:4 and 160–300 pmol PI(4,5)P_2_ 37:4 was spiked to the remaining pellet (which was kept on ice all the time). The pellet was resuspended in 375 µL chloroform/methanol/37% HCl (40:80:1, v/v/v), incubated at RT for 15 min and vortexed every 5 min for 30 s. After transferring the tube to ice, 125 µL cold chloroform and 225 µL cold 0.1 M HCl were added followed by 1 min vortexing. Phase separation was induced by centrifugation (6,500 × g, 2 min at 4 °C). The lower organic phase was immediately transferred to a new tube and kept on ice until further processing. For mass spectrometric analysis, an aliquot (10 µL) of the organic phase was diluted 1:2 with 50 mM ammonium acetate in chloroform/methanol (1:1, v/v).

*Mass spectrometry*: Before mass spectrometric analysis, lipid extracts were subjected to centrifugation (13,800 × g, 5 min at 4 °C) to remove debris. 20 µL of lipid samples were loaded into 96-well plates and sealed. Lipid infusion and ionization was achieved with the Triversa Nanomate® (Advion Biosciences) operated with the ChipSoft^TM^ Software (Advion Biosciences) under the following settings: sample infusion volume: 10 µL, volume of air to aspirate after sample: 1 µL, air gap before chip: enabled, aspiration delay: 0 s, pre-piercing: with mandrel, spray sensing: enabled, cooling temperature: 12 °C, gas pressure: 0.5 psi. The ionization voltage was set to 1.3–1.6 kV. For infusion in 10 mM ammonium acetate in methanol (DAG analysis) vent headspace was enabled and pre-wetting was done once. For infusion in 25 mM ammonium acetate in chloroform/methanol/H_2_O (PIP and PIP_2_ analysis) vent headspace was disabled and pre-wetting was performed two times.

Mass spectrometric analysis was performed with the QStar® Elite (Applied Biosystems) operated by Analyst® 2.0 and the AB Sciex QTrap® 5500 operated by Analyst® 1.5.1. Following, instrument-dependent settings were used (Table 2).

**Table 2:** Mass spectrometric settings used for lipid analysis.

	QStar® Elite	QTrap® 5500
Curtain Gas	10	10
CAD Gas	2	5
Operating pressure (torr)	2.5 × 10^−5^	1.6 × 10^−5^
Interface heater temperature IHT (°C)	40	40
Declustering potential (DP)	40	100
Focusing potential (FP)	200	-
Declustering potential 2 (DP2)	10	-
Entrance potential (EP)	-	7
Collision cell exit potential (CXP)	-	19
Detector (CEM)	2,500	2,100
Quadrupole resolution	Unit (Q1)	Unit (Q1 and Q3)

Product ion analysis and MPIS [29,33] was performed with the QStar® Elite. MPIS of DAG-specific MAG-H_2_O fragments (Table 3) was carried out at a CE of 25 eV. 44 MCA spectra for every precursor ion scan were acquired. Q1-scanning was done in the profile mode with a step size of 0.2 Da. The TOF mass range was set to *m/z* 250–400. Trapping of ions in Q2 (peak enhancement) was applied to *m/z* 327.3.

**Table 3:** DAG-specific MAG-H_2_O fragments used for MPIS analysis.

**MAG-H_2_O**	14:1	14:0	16:2	16:1	16:0	17:0	18:3	18:2	18:1
***m/z***	283.2	285.2	309.2	311.3	313.3	327.3	335.3	337.3	339.3
**MAG-H_2_O**	18:0	20:5	20:4	20:3	20:2	20:1	20:0	22:6	22:5
***m/z***	341.3	359.3	361.3	363.3	365.3	367.3	369.3	385.3	387.3

Scanning for neutral losses of 35 Da, 357 Da and 437 Da was achieved with the AB Sciex QTrap® 5500 at collision energies of 15 eV, 25 eV and 35 eV, respectively. Scanning was performed in the profile mode (step size 0.1 Da) at a scan rate of 200 Da/s. For every scan mode 100-500 MCA spectra were accumulated. Mass spectra were evaluated with the LipidView^TM^ Software Version 1.1 (AB Sciex). DAG, PIP and PIP_2_ amounts were normalized to PC, which was quantified in neutral extracts as described [62].

## 5. Conclusions

Here we present a mass spectrometric method to rapidly quantify signaling lipids down to the molecular species level. The method capitalizes on different extraction properties of DAGs and phosphoinositides allowing to efficiently recover by a quick two step-extraction procedure both lipid classes. Quantitative profiling is performed in the presence of internal standards by applying lipid class specific neutral loss (phosphoinositides) and multiple precursor ion scanning (DAGs). As a proof of principle, we monitor transient changes in the level of signaling lipids in human T cells before and after TCR stimulation. Quantitative profiling of signaling metabolites by nano-ESI MS/MS thus provides a valuable tool for future lipidomics studies in order to unravel mechanisms of lipid-based signaling events in health and disease.

## Supplementary Files

Supplementary File 1 DOC-Document (DOC, 104 KB)
