# Supplementary material for: Quantification of Signaling Lipids by Nano-Electrospray Ionization Tandem Mass Spectrometry (Nano-ESI MS/MS)
Source: Metabolites. 2012 Jan 16;2(1):57–76. doi: 10.3390/metabo2010057 (PMC3901191; doi:10.3390/metabo2010057)

**Supplementary Figure 1.** Effect of chain length and degree of unsaturation on detection of DAG species via neutral loss 35 (NL35) or multiple precursor ion scanning (MPIS). Indicated DAG species were quantified by NL35 or MPIS using DAG 17:0/17:0 as internal standard. In each case, DAG 28:0 amounts were set to 100%. Data are presented as mean values ± standard error of the mean (n = 12).


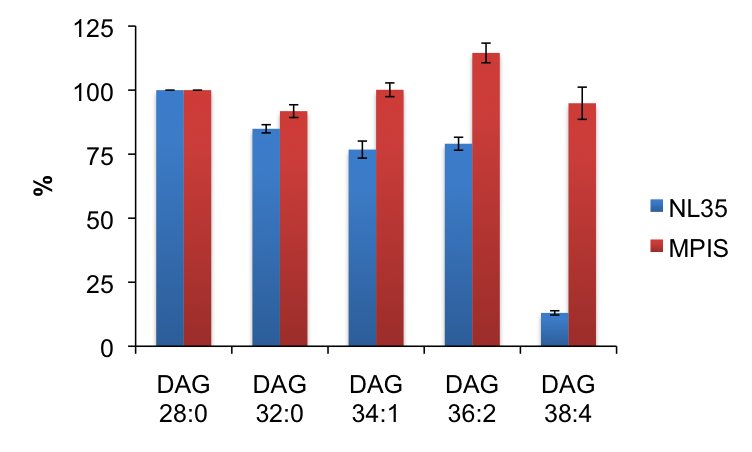

Supplement: Supplementary File 1 — DOC-Document (DOC, 104 KB) [file metabolites-02-00057-s001.doc]
